# Low Level of Haptoglobin in Lupus

**DOI:** 10.1177/2324709616689106

**Published:** 2017-01-01

**Authors:** Homa Timlin, Kirthi Machireddy, Michelle Petri

**Affiliations:** 1 Johns Hopkins University, Baltimore, MD, USA; 2 Philadelphia College of Osteopathic Medicine, Philadelphia, PA, USA

**Keywords:** haptoglobin, lupus, anemia

## Abstract

Haptoglobin levels are measured in systematic lupus erythematosus patients as part of the workup for anemia, with low levels indicating hemolysis. Haptoglobin is an acute phase protein. We present 2 lupus patients who were found to have low haptoglobin levels in the absence of other evidence of hemolysis.

## Case Presentation I

A 60-year-old African American woman was diagnosed with systematic lupus erythematosus (SLE) in December 1999, characterized by malar rash, arthritis, proteinuria, leukopenia, positive ANA (1:640, homogenous and speckled pattern), anti-Smith, anti-RNP, and low C3. Over the years her treatment included variable dose of steroids, hydroxychloroquine, azathioprine, and cyclophosphamide. During her routine follow-up visit, she was found to have mild alopecia. She was taking prednisone 5 mg and hydroxychloroquine 400 mg. The hematocrit was 36 (up from 33, 3 months prior to the visit), mean corpuscular volume 83, haptoglobin 9 mg/dL (normal 39-195 mg/dL), absolute reticulocyte count of 39 000/mm^3^ (normal 24 100-877 00), negative anti-dsDNA, C3 of 111 mg/dL (normal 79-152), C4 of 26 mg/dL (normal 12-42), normal platelet counts (216 000/mm^3^), normal urine protein creatinine ratio of 0.08 (normal 0.00-0.19 mg/g creatinine), normal liver function test (3 months prior and 3 months after the visit), and erythrocyte sedimentation rate was 24.

## Case Presentation II

A 42-year-old Caucasian man had SLE since 1992, manifested by malar rash, arthritis, serositis, proteinuria, leukopenia, lymphopenia, thrombocytopenia, positive immunology for ANA (1:640, homogenous/speckled pattern), anti-dsDNA, lupus anticoagulant, anticardiolipin, anti-Smith, anti-RNP, low C3, and C4. He was treated with variable dose of steroids, cyclophosphamide, mycophenolate mofetil, and hydroxychloroquine. During his follow-up appointment, he was found to have a new maculopapular rash, while on daily mycophenolate mofetil 1500 mg, hydroxychloroquine 400 mg, and prednisone 10 mg. The hematocrit was 30 (unchanged for 3 months prior and after visit), mean corpuscular volume 90, haptoglobin <6 mg/dL (normal 39-195 mg/dL), absolute reticulocyte count of 17 000/mm^3^ (normal 241 00-877 00), anti-dsDNA of 20, C3 of 49 mg/dL (normal 79-152), C4 of 10 mg/dL (normal 12-42), stable thrombocytopenia (109 00/mm^3^), normal urine protein creatinine ratio of 0.17 (normal 0.00-0.19 mg/g creatinine), normal liver function test (2 months prior and 3 months after the visit), and erythrocyte sedimentation rate was 13.

## Discussion

Haptoglobin is a plasma a2-sialoglycoprotein primarily synthesized in the liver, and to a lesser extent in other tissues including lung, skin, spleen, brain, intestine, arterial vessels, and kidney, and secreted in plasma. The primary role of haptoglobin is to bind free hemoglobin released during hemolysis or normal cell turnover and form haptoglobin-hemoglobin complexes. These complexes are then cleared and degraded by macrophages.^1^ The concentration in human plasma increases several fold in the occurrence of local or systemic inflammation, as a result of transcriptional activation of the haptoglobin gene by pro-inflammatory cytokines such as interleukin (IL)-1B, IL-6, and tumor necrosis factor. The concentration of serum haptoglobin can be low in the presence of liver disease, hemolytic anemia, ineffective erythropoiesis in myelodysplastic syndrome, hereditary ahaptoglobinemia, and with pregnancy and estrogen therapy. Haptoglobin levels can be increased in any disease in which the concentration of acute-phase reactants is increased, such as autoimmune diseases, infections, and malignancies.^2,3^

In SLE, the levels of haptoglobin are high especially when the disease is active. Haptoglobin level has been associated with cardiovascular disease in SLE.^4^ Low levels of haptoglobin are found, in SLE patients who have hemolytic anemia.

## Conclusion

We described 2 patients with SLE who had low haptoglobin levels in the absence of any other evidence of hemolysis. These 2 cases caution against using low haptoglobin alone as an indicator of hemolysis. Further research is needed to elucidate the mechanism underlying these findings.
