# Corrigendum

**DOI:** 10.1177/2324709617701789

**Published:** 2017-04-05

Timlin H, Machireddy K, Petri M. Low Level of Haptoglobin in Lupus. *J Investig Med High Impact Case Rep*. 2017;5(1):1-2. doi:10.1177/2324709616689106.

The affiliation of second author, Kirthi Machireddy, is incorrect. The correct author byline and affiliations are given below.

Homa Timlin, MD, MSc^1^, Kirthi Machireddy^2^, and Michelle Petri, MD, MPH^1^

^1^Johns Hopkins University, Baltimore, MD, USA

^2^University of the Sciences, Philadelphia, PA, USA

